# Anterior Interosseous Nerve to Pronator Quadratus Transfer to Restore Intrinsic Function: An Adjunct to Cubital Tunnel Decompression

**DOI:** 10.7759/cureus.85597

**Published:** 2025-06-09

**Authors:** Ahmed Elfaki, William M Nabulyato, Haneen Abed, Mohini Panikkar, David Izadi

**Affiliations:** 1 Plastic and Reconstructive Surgery, University Hospitals Coventry and Warwickshire, Coventry, GBR; 2 Orthopaedics and Trauma, Royal London Hospital, Barts Health NHS Trust, London, GBR

**Keywords:** ain, hemi-end-to-end, nerve, transfer, ulnar

## Abstract

Background

Injury or compression of the ulnar nerve impairs fine motor control, intrinsic hand function, and sensation in the small and ulnar side of the ring finger. Anterior interosseous nerve to pronator quadratus (AINPQ) transfer offers a potential solution, leveraging this expendable nerve to enhance the recovery of the ulnar nerve's motor function.

Method

This single-unit retrospective case series evaluates seven patients undergoing combined cubital tunnel decompression and AINPQ transfer for severe ulnar neuropathy. Data were collected from December 2020 to January 2023, including age, hand dominance, affected limb, and symptom duration (pain, sensory changes, motor weakness). The cohort comprised cases of both compressive and traumatic ulnar nerve injuries. Electrophysiological findings, intraoperative observations, postoperative assessments, and complications were recorded. Outcomes were measured using the Disabilities of the Arm, Shoulder, and Hand questionnaire at least six months postoperatively, with success defined as a ≥1 Medical Research Council (MRC) grade improvement or a score of >3 in thumb adduction, intrinsic function, or grip strength.

Results

All patients demonstrated a motor function improvement of ≥1 MRC grade following AINPQ transfer. The complication rate was low, with no reported cases of functional deterioration or infection, consistent with existing literature. One patient reported a painful scar, attributed to the cubital tunnel decompression procedure.

Conclusion

This study supports the adjunctive use of AINPQ with cubital tunnel decompression in severe ulnar nerve compression or injury cases. AINPQ shows potential in accelerating reinnervation and improving hand function within 12-14 months of symptom onset, though larger prospective studies are necessary for validating and refining patient selection criteria.

## Introduction

The ulnar nerve is essential for fine hand motor control and sensory input to the small and ulnar half of the ring finger. It can be compressed at various points from its origin in the spinal cord to its terminal branches in the hand, with cubital tunnel syndrome (CuTS) being the second most common compressive neuropathy, after carpal tunnel syndrome [1]. The clinical diagnosis is often supported by electrophysiological studies. Without timely intervention, CuTS can result in progressive sensory deficits, such as numbness, paraesthesia, and, in more severe cases, significant motor impairment due to intrinsic muscle denervation. This can lead to an intrinsic minus hand, characterised by muscle wasting and fibrosis, limiting hand function and causing a claw deformity [2].

Traditionally, tendon transfers have been used in end-stage disease to correct claw deformities by utilising expendable, unaffected tendons innervated by intact motor nerves, offering partial recovery to the complex, precise function of the intrinsic hand muscles [3].

Nerve transfers have gained prominence especially in cases where spontaneous ulnar nerve recovery is unlikely [4]. Timely reinnervation of intrinsic hand muscles is crucial, as motor endplates deteriorate after 18 months following denervation [5]. One promising strategy is the anterior interosseous nerve to pronator quadratus (AINPQ) transfer to augment the recovering motor component of the ulnar nerve [6,7]. This well-recognised method shortens the reinnervation distance, potentially preventing the irreversible degradation of motor endplates in the intrinsic hand muscles.

The "supercharged end-to-side" (SETS) transfer, popularised by Dr. Mackinnon, and the "hemi-end-to-end" (hemi-ETE) motor transfer are two established approaches, both aiming to reinnervate the hand's intrinsic muscles before complete motor endplate loss [6,8]. These procedures leverage the intact pronator teres muscle to compensate for the loss of PQ function without significant functional impairment. For clarity, when describing AINPQ, this will be in reference to the hemi-ETE transfer.

Our aim is to evaluate the hemi-ETE technique to determine the following: its effectiveness as a variant of the AINPQ transfer for ulnar motor nerve recovery, its utility in managing severely compromised intrinsic hand function, its applicability across various ulnar nerve pathologies (both traumatic and non-traumatic), and its potential complications.

## Materials and methods

This retrospective case series, conducted by a single surgeon in a single unit (University Hospitals Coventry and Warwickshire, Coventry, UK) between December 2020 and January 2023, reviewed outcomes of patients undergoing AINPQ transfer for severe ulnar neuropathy. Patients were identified from an electronic logbook, which included data from patient records, electrophysiology studies, operative notes, and postoperative follow-up appointments. Key demographic data collected included age, sex, hand dominance, affected limb, and the duration of symptoms such as pain, sensory changes, and motor weakness.

The inclusion criteria focused on patients presenting with severe ulnar neuropathy characterised by motor weakness, loss of power grip, weakened key pinch, or muscle wasting in the ulnar intrinsic muscles. Pathologies ranged from compressive neuropathies like CuTS to traumatic ulnar nerve injuries. Preoperative electrophysiological studies assessed nerve conduction velocities, pathology location, spontaneous and voluntary muscle activity, and sensory conduction. Median nerve functionality was also reviewed to evaluate the feasibility of AINPQ transfer.

Additional adjunctive procedures performed during the AINPQ transfer were documented. Intraoperative findings, postoperative reviews, and complications were recorded, specifically monitoring improvements in sensory disturbances, motor function, and power, which were assessed using the Medical Research Council (MRC) scale [9]. Patient-reported outcomes and functional improvements were evaluated through the Disabilities of the Arm, Shoulder, and Hand (DASH) questionnaire where a score of 0 indicated no disability and 100 represented severe functional impairment [10]. Due to the study's retrospective design, patients were asked to describe their function at least six months following surgery.

Outcome measures included improvement in MRC power grade in thumb adduction, indicating reinnervation of the adductor pollicis, one of the terminal ulnar nerve branches. An improvement of 1 MRC grade or a score of 3 or higher was considered a successful outcome. Patients were reviewed within three months post-surgery and followed for at least six months.

Technique 

We use the hemi-ETE AINPQ transfer, as described by George et al. [8], to address severe ulnar neuropathy. A cubital tunnel decompression, performed under regional anaesthesia with a high-arm tourniquet, is undertaken through an inverted curvilinear incision over the medial epicondyle. Guyon's canal is released to prevent any distal nerve compression from hindering nerve recovery. This involves Bruner incisions from the hypothenar eminence, extending proximally to the point where the dorsal sensory branch of the ulnar nerve is located, approximately 5-8 cm proximal to the distal wrist crease.

At this juncture, the ulnar nerve divides into three branches: dorsal sensory, motor, and volar sensory. Using intraoperative nerve stimulation with the Checkpoint biphasic nerve stimulator (Checkpoint Surgical, Independence, OH, USA) (initially 50 Hz and 0.5 mAmp increasing to 1-2 mAmp if necessary), the motor branch of the ulnar nerve is identified [11,12]. The fascicular topography of the motor branch at this level remains complex and becomes more organised as it reaches its distal targets.

**Figure 1:**
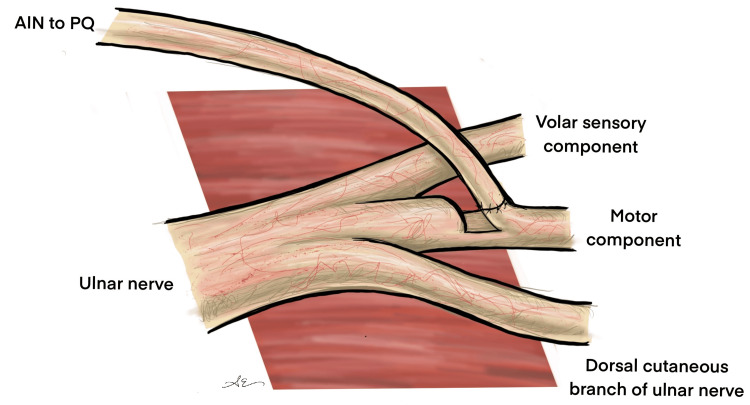
Schematic representation of the hemi-ETE AINPQ transfer after intrafascicular dissection of the volar sensory and motor components of the ulnar nerve Image Credits: Ahmed Elfaki Hemi-ETE: hemi-end-to-end; AINPQ: anterior interosseous nerve to pronator quadratus

The hemi-ETE AINPQ transfer involves harvesting 50% of the ulnar motor nerve for the reinnervation of ulnar intrinsic motor muscles. This reinnervation serves as a "babysitter" for the motor endplates, preventing their degradation while awaiting native ulnar nerve recovery following cubital tunnel decompression (Figure 1).

**Figure 2:**
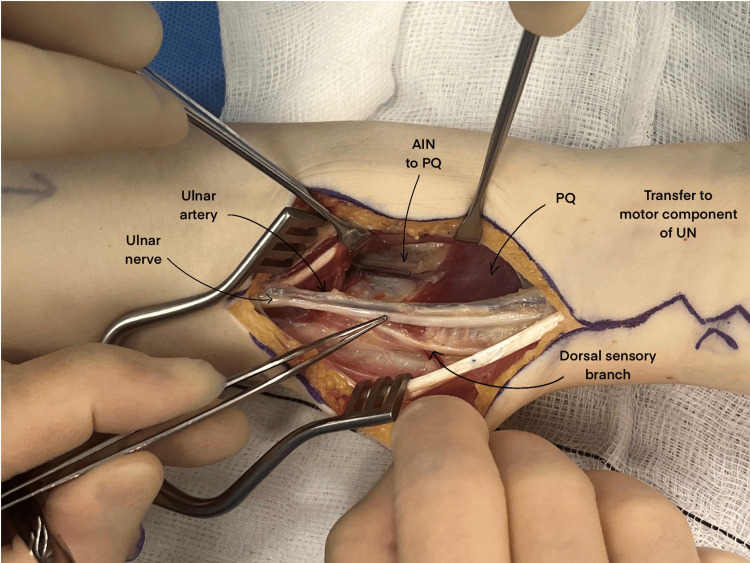
Incision to locate the AINPQ branch prior to its dissection with relevant anatomy demonstrated AINPQ: anterior interosseous nerve to pronator quadratus; UN: ulnar nerve

The AINPQ branch is identified in the distal volar forearm by locating the PQ and its transverse fibres (Figure 2). The AIN enters the PQ in the midline and can extend for up to 3 cm before branching. Additional length is achieved by dissecting through the PQ. The nerve transfer is performed tension-free in wrist extension to maximise the chances of successful recovery.

**Figure 3:**
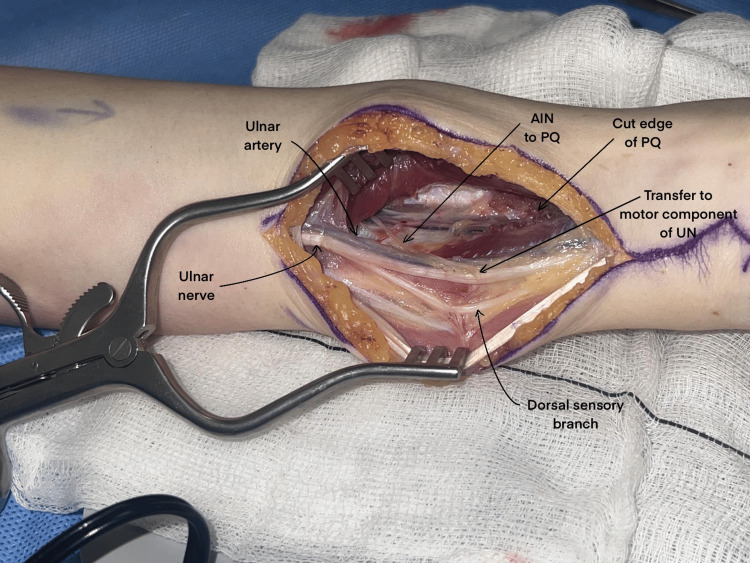
The AINPQ branch has been dissected out and transposed to the motor component of the UN prior to nerve transfer AINPQ: anterior interosseous nerve to pronator quadratus; UN: ulnar nerve

Fifty percent of the ulnar motor nerve is then divided distal to the branching of the dorsal cutaneous sensory branch, followed by the intrafascicular dissection of the distal half, which is reflected towards the AINPQ for coaptation (Figure 3). The transfer is secured using interrupted 8-0 nylon sutures and Tisseel fibrin glue [13]. After closure, the patient is placed in a bulky bandage and commences hand therapy 3-5 days postoperatively. Patients are instructed by hand therapists on performing pronation and resisted pronation exercises to promote ulnar nerve reinnervation [1,14].

## Results

Seven patients (M:F, 6:1) underwent a combined cubital tunnel release and AINPQ transfer. The mean age was 45 (range 16-76). Five were right-hand dominant, one was left-hand dominant, and one was ambidextrous. Two patients were smokers. The duration of symptoms preceding surgery ranged from 147 to 431 days (median 322 days). Table 1 summarises the results below. The underlying pathologies included compressive ulnar neuropathy (four), complete ulnar nerve transection (one), an infraclavicular brachial plexus injury (one), and ulnar neuropathy following an arteriovenous malformation excision in the cervical spine (one).

**Table 1:** Patient demographics, procedure details, and outcomes Bold is used to highlight that this patient had recurrent CuTS. CuTS: cubital tunnel syndrome; hemi-ETE: hemi-end-to-end; EIP: extensor indicis proprius; APB: abductor pollicis brevis; AINPQ: anterior interosseous nerve to pronator quadratus

Patient no.	Age (in years)	Gender	Hand dominance	Side of surgery	Smoker	Mechanism	Symptom duration in days (months)	Procedures	Nerve conduction class	DASH score post-op	Finger abduction/adduction	Clawing	Grip strength	Thumb adduction	Functional improvement	Complications	Follow-up period (months)
1	58	M	Ambidextrous	Right	Non-smoker	Compression (CuTS)	147 (5)	Hemi-ETE AINPQ transfer. Cubital tunnel decompression. Guyon's canal decompression	Very severe	28	3/5 (was 2/5)	Resolved (36 days)	Improved	4/5 (improved from 1/5 in 7 weeks)	Thumb adduction 5/5	Nil	6
2	76	M	Right	Right	Non-smoker	Compression (CuTS)	377 (12)	Hemi-ETE AINPQ transfer. Cubital tunnel decompression. Guyon's canal decompression	Very severe	1	5/5 (was 2/5)	Resolved (29 days)	Improved (from 13.9 kg to 21.9 kg in 9 weeks)	5/5	Near-normal function	Nil	6
3	55	M	Right	Right	Smoker	Compression (recurrent CuTS)	431 (14)	Hemi-ETE AINPQ transfer. Revision cubital tunnel decompression. Guyon's canal decompression	Severe	8	4/5 (was 1/5)	Slight improvement	Mild improvement (34 kg)	5/5	Improved strength in ring and little fingers. Returned to work	Cold intolerance	6
4	43	M	Right	Right	Smoker	Compression (CuTS) following fall/osteoarthritis	278 (9)	Hemi-ETE AINPQ transfer. Cubital tunnel decompression	Severe	16	5/5	Resolved	Improved 5/5	5/5	Tingling (no improvement). Returned to work	Elbow scar discomfort (treated with steroid injections)	36
5	47	M	Left	Right	Non-smoker	Infraclavicular brachial plexus palsy (shoulder dislocation)	322 (10)	Hemi-ETE AINPQ transfer. Cubital tunnel decompression. Carpal tunnel decompression. EIP to APB opponensplasty	Severe	12	5/5 (was 0/5)	Improved	Improved 5/5 (from 18 kg to 40 kg)	5/5 (was 1/5)	Sensation improved. Returned to work	Nil	11
6	24	M	Right	Left	Non-smoker	Repaired complete transection at elbow level	288 (9)	Hemi-ETE AINPQ transfer. Cubital tunnel release and nerve transposition. Excision of neuroma and cable nerve graft	Severe	48	1/5 (was 0/5)	Improved		Nil	Dismissed from work	Nil	6
7	16	F	Right	Right	Non-smoker	Excision of arteriovenous malformation of the cervical spine	345 (11.5)	Hemi-ETE AINPQ transfer. Cubital tunnel release	Not performed	8	3/5	Improved		0/5	Increased hypothenar muscle bulk, improved sensation	Nil	6

The indication for surgery was for patients with severe ulnar neuropathy with intrinsic muscle wasting within one year of symptom onset and no signs of recovery. Intact motor endplates in the first dorsal interosseous (FDI) muscle were confirmed by electromyography (EMG).

All patients showed clinical signs of intrinsic muscle wasting and motor weakness. One patient had previously undergone cubital tunnel release four years prior and required revision surgery. Nerve conduction studies confirmed severe or very severe ulnar neuropathy in six patients.

Postoperatively, all patients reported motor improvement. Six patients had an increase in their MRC grade of at least 1 for power grip. Thumb adduction improved in five patients. One patient, a musician who had lost the ability to play the trumpet due to intrinsic muscle weakness, regained partial function within two months and achieved near-complete hand function within six months. 

Patient 3 initially reported no improvement in power and experienced worsening numbness and sensory loss. Of note, he had the longest symptom duration and delayed presentation and previous cubital tunnel decompression. One patient also reported mild scar discomfort, while the revision cubital tunnel patient noted persistent cold intolerance in the affected hand.

All patients completed DASH questionnaires postoperatively. Patient 6 had the worst outcome due to a complete nerve transection at the level of the elbow and had the highest DASH score (indicating a lower functional recovery). His transfer was nine months post-injury and had additional morbidity due to a neuroma at the previous repair site, requiring excision and nerve graft. Unfortunately, he was unable to return to work due to dismissal. The mean follow-up was 11 months, with one patient followed for up to three years. Table 2 summarises the results of the neurophysiology studies in relation to their postoperative DASH score.

**Table 2:** Neurophysiology findings and DASH scores IA: insertional activity; fib: fibrillations: PSW: positive sharp waves; NR: not recordable; FDI/ADM: first dorsal interosseous/abductor digiti minimi; DASH: Disabilities of the Arm, Shoulder, and Hand

Patient no.	Nerve conduction class	FDI				ADM				FDI/ADM amplitude	Ulnar nerve conduction velocity (ms)	DASH score	Comments
		IA	Fib	PSW	Amplitude (mV)	IA	Fib	PSW	Amplitude (mV)				
1	Very severe	N	1+	1+	0.8	N	1+	1+	4.2	Reduced both	39-48	28	ADM and FDI amplitude reduced, no latency
2	Very severe	NR	NR	NR	-	NR	NR	NR	0.2	Reduced ADM	NR	1	Right ulnar motor response from ADM and FDI muscles barely recordable
3	Severe	N	N	N	0.9	N	N	N	0.5	Reduced both	33-50	8	Chronic active denervation changes in the right ADM and FDI
4	Severe	N	2+	2+	-	N	N	N	2.1	Reduced ADM	54	16	Active denervation potentials noted in the right FDI. No volitional activity in FDI. Recruitment in the right flexor digitorum profundus and ADM is significantly reduced
5	Severe		1+	3+	0.46	N	N	N	1.26	Reduced both	50	12	No voluntary activity in FDI
6	Very severe	NR	NR	NR		NR	NR	NR		NR	NR	48	ADM/FDI NR
7	Not performed preoperatively												

## Discussion

Ulnar nerve dysfunction, particularly CuTS, can be debilitating, causing sensory disturbances and progressive muscle weakness and atrophy with varying severity as described by McGowan [15] and Dellon [16]. Surgical decompression of the cubital tunnel has a failure rate of 2.4-17% due to the incomplete reinnervation of the ulnar motor intrinsics [17]. This study aimed to determine if AINPQ transfer to the ulnar nerve can restore intrinsic hand function in severe cases where decompression alone is insufficient.

The study goals are the following: (1) assess if AINPQ transfer restores intrinsic hand function, (2) determine if recovery is attributable to the nerve transfer, (3) establish the timeline for regaining function, and (4) identify complications associated with the technique.

Our primary indication for hemi-ETE is for patients with severe ulnar neuropathy (intrinsic muscle wasting) within one year of symptom onset with intact motor endplates and no signs of recovery either clinically or via neurophysiological studies.

All patients reported improvements in hand function (grip strength, key pinch, DASH scores) within 2-3 months post-surgery, suggesting the nerve transfer was effective in hastening reinnervation compared to decompression alone, which could take up to two years. The complication profile was low, with no infections and only one case of mild scar discomfort.

A hemi-ETE transfer connects the AINPQ to 50% of the ulnar motor nerve and preserves the continuity of the ulnar nerve for potential recovery from cubital tunnel decompression while offering early reinnervation. This differs from traditional end-to-end or end-to-side nerve transfers, which may sacrifice the recipient nerve or delay intrinsic muscle function restoration.

Most of our patients (6/7) demonstrated some intrinsic muscle recovery within six months, indicating the AINPQ transfer's contribution. One patient (Table 2, Patient 1) with the shortest symptom duration and higher compound muscle action potential (CMAP) amplitude (which was 4.2 mV) reported less initial symptomatic improvement compared to others with lower CMAP values (0.2-2.1 mV) and longer symptom duration (9-14 months), who reported significant improvement. This disparity between CMAP values and clinical outcomes highlights the variability in patient reporting and the limitations of relying solely on neurophysiological measures for functional assessment.

The average symptom duration before surgery was 10 months (range 5-14), which might have allowed for native nerve recovery and maintained the motor endplates to allow cubital tunnel decompression the time to be effective [5]. There was no correlation between symptom duration and improvement in intrinsic muscle recovery, to similar findings by Dengler et al. [1]. There was also no correlation with age or CMAP values.

Literature review

There are mixed results regarding the success of nerve transfers in CuTS. Dengler et al. reported a 7% failure rate and identified age as a significant predictor of poor outcomes when retrospectively analysing 42 patients' SETS AINPQ transfers [1]. Interestingly, the duration of symptoms and the presence of motor endplates did not correlate with recovery. Davidge et al. similarly demonstrated that 60% of patients recovered intrinsic hand function within three months due to the reversal of ischaemia and remyelination following SETS transfer [7].

Our study observed recovery within a similar timeframe of 2-3 months, suggesting that the AINPQ transfer likely played a critical role in accelerating recovery by delivering regenerating axons directly to the ulnar motor nerve, shortening the reinnervation distance.

Baltzer et al. reported similar findings in a smaller cohort, where four out of six patients with compressive aetiology demonstrated intrinsic muscle recovery within 2.3 months following a SETS transfer [18]. Animal studies support that nerve transfers can reinnervate muscle to regain function [19]. George et al. achieved an 81.3% success rate in traumatic high ulnar nerve injuries using various types of nerve transfers, including end-to-end and SETS [8].

End-to-end transfers generally maximise axonal regeneration by providing a direct pathway for donor axons to reach their targets, but this sacrifices the donor nerve [20,21]. The hemi-ETE transfer (George et al. [8]) offers the advantage of partial preservation of the donor nerve while still facilitating early reinnervation of the ulnar intrinsic muscles. This supports the notion that preserving some continuity in the ulnar nerve can allow for a second wave of native recovery post-decompression.

End-to-side transfers maintain the donor nerve's continuity by creating an epi- or perineural window to connect the recipient nerve in order to preserve its initial function. In a reverse end-to-side transfer, the donor nerve is sacrificed and sutured to a window in the recipient nerve.

The topography of the ulnar nerve is well-documented, with its motor component consistently sandwiched between the dorsal and volar sensory fibres [22,23]. This allows for the precise coaptation of the AINPQ to the motor fibres with minimal morbidity. The share of motor axons increases and becomes more organised distally as they enter the motor component of the ulnar nerve [24] to reach their end-muscular targets. Anastomosing the AINPQ as proximally onto the ulnar motor component can potentially maximise regeneration to give a better generalised restoration of the intrinsics.

Limitations

The study's retrospective nature and reliance on patient-reported outcomes via the DASH questionnaire introduce recall bias. The cohort was small, and inconsistent nerve conduction studies limit our ability to track neurophysiological recovery. Future studies would benefit from prospective study designs, larger sample sizes, and routine electrophysiological testing. Nerve conduction studies postoperatively may have been beneficial in assessing for changes in latency, conduction velocity, or amplitudes, but present challenges due to the inconsistent documentation of the contralateral "good" side for comparison and user-dependent variability.

Challenges 

Adjunct procedures present the challenge of identifying what the true underlying mechanism is for recovery. Valid arguments are that the cubital tunnel decompression reverses chronic ischaemia to the ulnar nerve, which subsequently permits remyelination to aid nerve recovery and return to function which are time-dependent processes, and that the role of AINPQ is debatable.

Nerve recovery following injury occurs at a rate of 1 mm per day [25]. Given that patients in our series as well as the existing literature demonstrated functional improvements or signs of intrinsic recovery within three months, we suspect that the AINPQ transfer's role in recovery is promising and ought to be examined further. Recovery at this rate is supportive of increased distal axonal regeneration rather than reliance on proximal decompression and subsequent remyelination.

Furthermore, the literature suggests that decompression alone can take up to 4.5 years for functional recovery, as described by Matsuzaki et al., with electrophysiological improvement beyond two years [26]. However, the study did not specifically comment on any recovery of intrinsic muscle bulk.

We note that one patient in this series suffered an infraclavicular brachial plexus injury following a shoulder dislocation which would normally exclude them from a study like this. The rationale for their inclusion was due to their improving symptoms at the time of presentation and their near-normal recovery clinically prior to AIN to ulnar nerve transfer.

## Conclusions

We observe that AINPQ transfer combined with cubital tunnel decompression for severe ulnar nerve compression/injury shows promise in hastening reinnervation and improving hand function within 12-14 months of symptom onset. While this technique is yet to be widely adopted, this study can aid in forming the basis for larger prospective multicentre trials with controls to validate these findings and refine patient selection criteria. Nonetheless, the low complication rate and potential for early functional recovery make this technique a valuable option for improving outcomes in severe CuTS.
